# Potential Roles of Sestrin2 in Alzheimer’s Disease: Antioxidation, Autophagy Promotion, and Beyond

**DOI:** 10.3390/biomedicines9101308

**Published:** 2021-09-24

**Authors:** Shang-Der Chen, Jenq-Lin Yang, Yi-Heng Hsieh, Tsu-Kung Lin, Yi-Chun Lin, A-Ching Chao, Ding-I Yang

**Affiliations:** 1 Department of Neurology, Kaohsiung Chang Gung Memorial Hospital, Kaohsiung City 83301, Taiwan; chensd@adm.cgmh.org.tw (S.-D.C.); tklin@adm.cgmh.org.tw (T.-K.L.); 2 Institute for Translation Research in Biomedicine, Kaohsiung Chang Gung Memorial Hospital, Kaohsiung City 83301, Taiwan; jyang@adm.cgmh.org.tw; 3 Institute of Brain Science, National Yang Ming Chiao Tung University, Taipei City 11221, Taiwan; p400540226@gmail.com; 4 College of Medicine, Chang Gung University, Taoyuan City 33302, Taiwan; 5 Center for Mitochondrial Research and Medicine, Kaohsiung Chang Gung Memorial Hospital, Chang Gung University College of Medicine, Kaohsiung City 80708, Taiwan; 6 Department of Neurology, Taipei City Hospital, Taipei City 10629, Taiwan; DAB16@tpech.gov.tw; 7 Department of Neurology, College of Medicine, Kaohsiung Medical University, Kaohsiung City 80708, Taiwan; 8 Department of Neurology, Kaohsiung Medical University Hospital, Kaohsiung City 80756, Taiwan; 9 Brain Research Center, National Yang Ming Chiao Tung University, Taipei City 11221, Taiwan

**Keywords:** Alzheimer’s disease, autophagy, mTOR, oxidative stress, sestrin2

## Abstract

Alzheimer’s disease (AD) is the most common age-related neurodegenerative disease. It presents with progressive memory loss, worsens cognitive functions to the point of disability, and causes heavy socioeconomic burdens to patients, their families, and society as a whole. The underlying pathogenic mechanisms of AD are complex and may involve excitotoxicity, excessive generation of reactive oxygen species (ROS), aberrant cell cycle reentry, impaired mitochondrial function, and DNA damage. Up to now, there is no effective treatment available for AD, and it is therefore urgent to develop an effective therapeutic regimen for this devastating disease. Sestrin2, belonging to the sestrin family, can counteract oxidative stress, reduce activity of the mammalian/mechanistic target of rapamycin (mTOR), and improve cell survival. It may therefore play a crucial role in neurodegenerative diseases like AD. However, only limited studies of sestrin2 and AD have been conducted up to now. In this article, we discuss current experimental evidence to demonstrate the potential roles of sestrin2 in treating neurodegenerative diseases, focusing specifically on AD. Strategies for augmenting sestrin2 expression may strengthen neurons, adapting them to stressful conditions through counteracting oxidative stress, and may also adjust the autophagy process, these two effects together conferring neuronal resistance in cases of AD.

## 1. Introduction

Patients with age-related neurodegenerative diseases usually present with a relentlessly deteriorating clinical course. Worst of all, the lack of effective treatment results in heavy socioeconomic burdens to patients, family, and the whole of society [1,2,3]. Alzheimer’s disease (AD), a type of dementia with progressive memory loss and declined cognitive functions, is the most common neurodegenerative disease in the elderly. Based on the information from the World Health Organization (WHO), approximately 50 million people suffer from dementia worldwide, and nearly 10 million new cases are added every year, making the disease one of the main causes of disability and dependence. AD may account for 60–70% of all dementia cases (https://www.who.int/news-room/fact-sheets/detail/dementia, accessed on 21 September 2020). According to “2021 Alzheimer’s disease facts and figures”, in the USA [4], approximately 6.2 million senior Americans over 65 years old have AD. By 2060, with a steep projected increase, the number of AD patients may rise to 13.8 million. Data revealed that, from 2000 to 2019, deaths resulting from human immunodeficiency virus (HIV), heart disease, and stroke declined, while deaths from AD increased more than 145% [4]. The total healthcare costs in 2020 are approximated at $305 billion and are expected to increase to more than $1 trillion as the population ages [5]. It is crucial to delay, reduce, or prevent the occurrence of disability from AD and lessen the heavy burden it places on society.

The major pathological hallmarks of AD brains are gross atrophy of the brain, as well microscopically observable senile plaques and neurofibrillary tangles (NFTs) [6,7,8]. Senile plaques are extracellular structures mainly composed of insoluble deposits of amyloid-beta peptide (Aβ), a peptide fragment of 39–43 amino acids derived from sequential cleavage of the transmembrane protein amyloid precursor protein (APP) by β- and γ-secretase [9,10,11,12]. Newly synthesized full-length APP is transported from the endoplasmic reticulum (ER) to the Golgi apparatus (GA)/*trans*-Golgi network (TGN) for further protein processing and maturation. The acidic environment (pH = 6.0–6.5) in the TGN or the late GA is optimal for the activity of many processing enzymes, including BACE1. The full-length APP delivered to the plasma membrane may be subjected to non-amyloidogenic cleavage by α- and then γ-secretase to release the soluble APP-alpha (sAPPα), the p3 fragment, and the APP intracellular domain (AICD). Alternatively, a portion of the full-length APP may also be endocytosed into early endosomes and possibly rerouted to the acidic recycling endosomes (REs), where BACE1 resides, to produce Aβ [13]. In addition, extracellular Aβ can also be taken up through receptor binding and subsequently internalized, thereby leading to its accumulation within various intracellular compartments, including endosomes, multivesicular bodies (MVBs), lysosomes, mitochondria, the ER, the TGN, and cytosol [14].

Aβ can induce neurotoxicity through various mechanisms, such as excitotoxicity [15], excessive generation of reactive oxygen species (ROS) [16], aberrant cell cycle reentry [17,18], impaired mitochondrial function [19], and DNA damage [20], all of these mechanisms together contributing to neuronal damage or even death. Moreover, Aβ can also alter gene transcription [19], and thereby affect protein expression, which may influence the survival or death of neuronal cells in AD-related pathophysiology.

Maintenance of neuronal functions depends on axonal transport of proteins, organelles, and vesicles from the soma to the nerve terminals [21]. Going the other way, neurotrophic factors, including the members of the neurotrophin family, secreted from post-synaptic targets must be transmitted retrogradely from nerve terminals via axonal transport back to the soma [22]. Thus, failure of axonal transport may contribute to neuronal death. As a microtubule-binding protein important for microtubule assembly and stabilization, hyperphosphorylation of tau compromises its biological functions and destabilizes the structures of microtubules, and is accompanied by disturbance to axonal transport [23]. Furthermore, increasing evidence suggests that Aβ may also disrupt axonal transport and contribute to AD pathophysiology [21].

**Figure 1:**
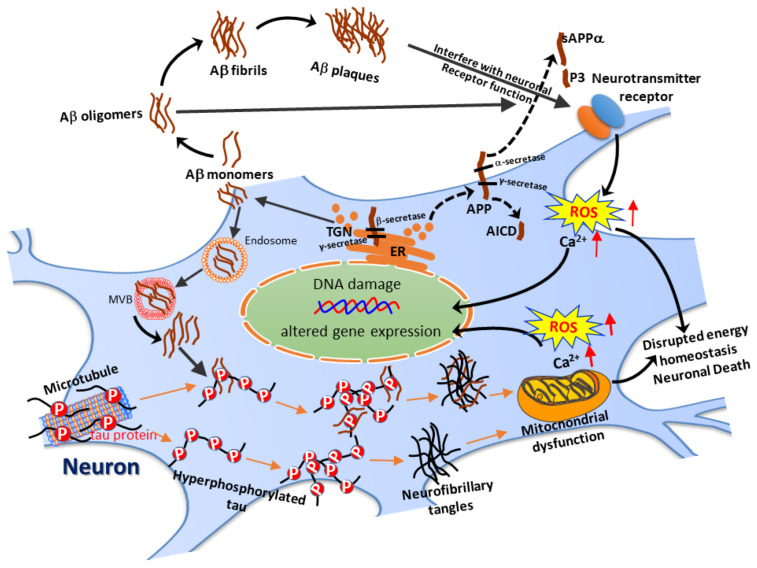
The cartoon diagram demonstrates the pathogenic processes of amyloid-beta peptide (Aβ) and tau protein. Through the amyloidogenic pathway, the full-length amyloid precursor protein (APP) is sequentially cleaved by β-secretase (encoded by beta-site amyloid precursor protein cleaving enzyme-1 or BACE1) and γ-secretase to generate Aβ. Newly synthesized APP is transported from the endoplasmic reticulum (ER) to the Golgi apparatus (GA) for protein maturation. The acidic pH in the trans-Golgi network (TGN) or the late GA is optimal for BACE1 activity, with production of secreted Aβ; the sequential amyloidogenic cleavages of full-length APP by β- and γ-secretase also generate soluble APP-beta (sAPPβ) and the APP intracellular domain (AICD), though these are not depicted in the diagram. A portion of the full-length APP reaching the plasma membrane may be subjected to the non-amyloidogenic cleavage by α- and then γ-secretase to release the soluble APP-alpha (sAPPα), the p3 fragment, and the AICD. Another portion of the full-length APP may also be endocytosed into early endosomes and possibly be rerouted to the acidic recycling endosomes (REs; not depicted), where BACE1 resides, for intracellular production of Aβ. Furthermore, extracellular Aβ can also be taken up through receptor binding and subsequent internalization, resulting in its accumulation within various intracellular compartments, including endosomes, multivesicular bodies (MVBs), and mitochondria (not depicted). The extracellular Aβ monomers aggregate into oligomers and then into fibrils, eventually forming senile plaques. Tau protein is a microtubule-binding protein, which is hyperphosphorylated in AD neurons. The phosphor-tau monomer may also aggregate into tau oligomers and, finally, into neurofibrillary tangles (NFTs). The intraneuronal Aβ species also oligomerize or even mix with tau proteins to form mixed aggregates. The extracellular senile plaques, the extracellular and intraneuronal Aβ oligomers, as well as tau oligomers and NFTs, together lead to excessive production of reactive oxygen species (ROS), Ca^2+^ overload, mitochondrial dysfunction, and disrupted energy homeostasis, ultimately causing neuronal death. In addition to those pictured above, other pathogenic mechanisms are not demonstrated in this figure due to limited space. For example, loss of tau binding destabilizes microtubules, thus compromising anterograde axonal transport of proteins, mitochondria, and vesicles from soma to the nerve terminals, which may negatively impact nerve transmission. Conversely, neurotrophic factors, especially neurotrophins, secreted from target cells also fail to be retrogradely transported from the nerve terminal back to the soma to nourish the neurons, also leading to neuronal demise. Please see the text for more details.

It was proposed two decades ago that fibrils may not be the only toxic form of Aβ; small oligomers of Aβ, or Aβ-derived diffusible ligand (ADDL), and Aβ protofibrils may also have potent neurotoxicity [24]. Like Aβ oligomers, tau oligomers formed during the early stages of aggregation are also pathologically relevant to the loss of neurons and behavioral impairments in several neurodegenerative disorders called tauopathies, the most common of which is AD [25]. In addition to the aggregation of extracellular amyloid plaques, emerging evidence has revealed the crucial role of intraneuronal amyloid species (iAβs) which can appear in the membrane or the lumen of late endosomes and precede further aggregation, eventually accumulating inside the endosome or endolysosome [26,27]. It was also noted that, besides the extracellular aggregation of homologous Aβ species, cross-seeding of different amyloid proteins, or even between different misfolded proteins, such as Aβs and tau, may be biologically significant, and even critical in the progression of AD [28]. Apart from cross-seeding, crosstalk between Aβ and tau may also play a vital role contributing to AD pathogenesis. For example, Aβ has been shown to trigger alternative splicing of tau isoforms via glycogen synthase kinase-3beta (GSK-3β), making tau more susceptible to hyperphosphorylation [29,30]. Overall, these effects could further aggravate aberrant cellular signaling, induce excessive tau phosphorylation, worsen toxic tau accumulation, and lead to synapto/neurotoxic effects [26]. A simplified cartoon summarizing the pathogenic mechanisms of AD is shown in Figure 1, below.

Sestrins, including sestrin1, sestrin2, and sestrin3, belong to a group of highly evolutionarily conserved proteins in mammalian cells, and may play a crucial role in stressful conditions, such as oxidative stress, hypoxia, and DNA damage [31,32,33,34]. While the structures of sestrin1 and sestrin3 await further elucidation, the essential characteristics of sestrin2 have been gradually revealed in recent years [35,36]. Three distinctive functional sites were identified, which are critical for inhibition of ROS production, modulation of the mammalian/mechanistic target of rapamycin (mTOR) complex 1 (mTORC1), and for leucine-binding [35,36]. Inhibiting either ROS for antioxidation or mTORC1 for autophagy promotion may attenuate degenerative processes associated with aging [35]. Therefore, sestrins may possess two beneficial effects that are pivotal for anti-aging [37,38].

Despite the potential effect of sestrins on age-related neurological disorders, only quite limited studies about AD have been reported. We have shown in a previous study that sestrin2 was induced by Aβ in primary rat cortical neurons and an increased expression of sestrin2 was also found in the cortices of 1-year-old AD transgenic mice [39]. We also showed that sestrin2 functions as an endogenous protective mediator against Aβ-induced neurotoxicity, in part through enhancement of autophagy activity [39]. In another recent study, we further demonstrated that Aβ-induced sestrin2 expression contributes to antioxidative activity in neurons; furthermore, Aβ induction of sestrin2 is at least partly mediated by the activation of transcription factors NF-κB and p53 [40]. In this review article, we discuss recent progress in revealing the underlying molecular mechanisms concerning the sestrin2-mediated protective effects against neuronal dysfunction in AD. Better understanding of the potential novel pathway in AD may guide further research into developing effective therapeutic regimens in the future. Finding the way to augmenting sestrin2 expression may have significant clinical implications, especially in treating many devastating neurodegenerative diseases, including AD.

## 2. The Biological Roles of Sestrin2

Sestrins, including sestrin1, sestrin2, and sestrin3, belong to a gene family and function as stress-inducible proteins that affect metabolism through perceiving nutrient status and redox level in living organisms. Sestrin1 (also known as PA26) was initially discovered in human Saos-2 osteosarcoma cells as one of the p53-induced transcripts and was mapped to chromosome 6q21 through a differential display screening [34,41]. Sestrin1 is ubiquitously expressed in most tissues, including lung, kidney, pancreas, skeletal muscle, and brain tissues [33], and it can be activated under oxidative stress and irradiation in a p53-dependent fashion [34,42]. Sestrin2 (also known as Hi95), located in chromosome 1p35.3, was first discovered in glioblastoma cells under prolonged hypoxia and its transcription was found to be increased following DNA damage [33]. Later, it was noted that sestrin1 and sestrin2, through activating the AMP-dependent kinase (AMPK) pathway, may affect tuberous sclerosis complex 2 (TSC2) expression to inhibit mTOR-mediated cell over-proliferation [43]. Sestrin3, located in chromosome 11q21, was identified from database mining of the PA26-related gene family [32,33]. mRNA expression of these sestrin genes is presented diffusely during mouse embryogenesis and also in adult tissues at various levels [32]. Sestrin1 is robustly expressed in the brain, heart, liver, and skeletal muscle; sestrin2 is expressed more in the kidney, leucocytes, lungs, and liver; sestrin3 is expressed at higher levels in the brain, kidney, small intestine, and skeletal muscle [32,34,44].

**Figure 2:**
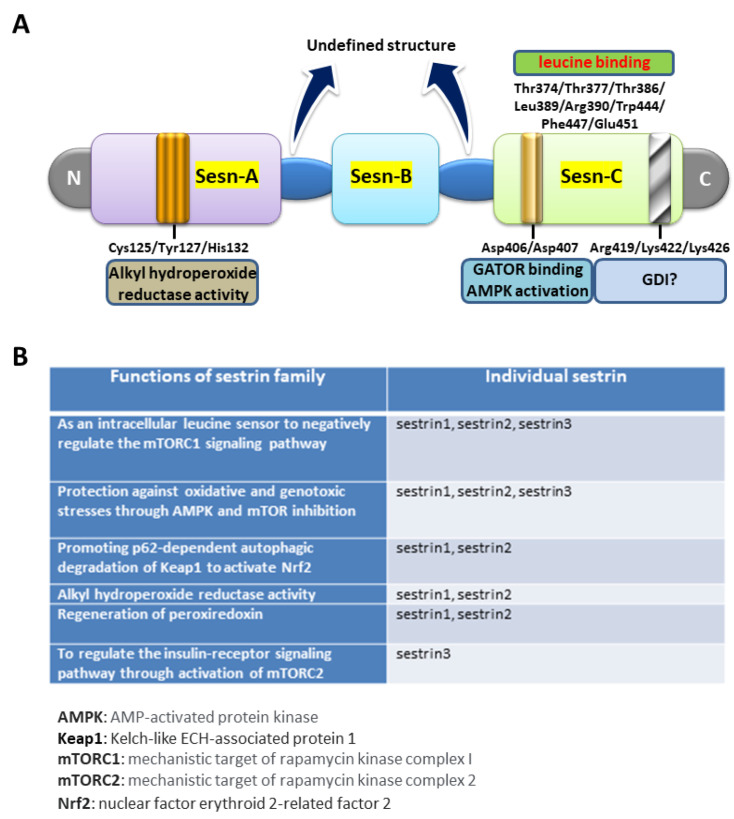
The structural and functional domains as well as the biological functions of three sestrin members. (**A**) The strip diagram illustrates the three major structural domains (Sesn-A, Sesn-B, and Sesn-C). Cys125/Tyr127/His132, located within the Sesn-A domain, is critical for alkyl hydroperoxidase activity. The Asp406/Asp407 residues, the so-called “DD motif”, located within Sesn-C are vital for GATOR2 binding and AMPK activation, both contributing to mTORC1 suppression. The leucine binding pocket spanning from Thr374 to Glu451 in the Sesn-C is also important for amino acid sensing and mTOR regulation. The guanosine nucleotide dissociation inhibition (GDI) domain containing Arg419/Lys422/Lys426 is also shown in Sesn-C. Based on the crystal structure, however, whether these amino acid residues are critical for GDI functions remains questionable. All the information was based on Kim et al., 2015 [35] and Saxton et al., 2016 [36]. (**B**) Potential biological functions of three sestrins are listed. Information was derived from UniProt (https://www.uniprot.org) for human sestrin1 [UniProtKB-Q9Y6P5 (SESN1_HUMAN)], human sestrin2 [UniProtKB-P58004 (SESN2_HUMAN), human sestrin3 [UniProtKB-P58005 (SESN3_HUMAN)], and mouse sestrin3 [UniProtKB- Q9CYP7 (SESN3_MOUSE)].

It has been revealed that the crystal structure of human sestrin2 (hSesn2) has distinct globular subdomains, each possessing separate functions [35]. As shown below in Figure 2A, the N-terminal domain (Sesn-A) diminishes alkyl hydroperoxide radicals through the helix-turn-helix oxidoreductase motif. Mutations of Cys125, His132, and Tyr127, which are, respectively, the catalytic cysteine, the residue critical for the conserved proton relay system, and the residue potentially involved in the catalytic process, reduce this redox activity. The C-terminal domain (Sesn-C) of hSesn2, whose sequence is highly conserved across the sestrin family, has lost its antioxidant activity but acquired another important function in mTORC1 inhibition via physical association with GTPase-activating protein activity toward the Rags-2 (GATOR2) complex, in which process Asp406 and Asp407 (the DD motif) are vital. Furthermore, the DD motif is involved in activation of AMP-dependent protein kinase (AMPK), which is also important for mTORC1 inhibition. Besides GATOR2 binding and AMPK activation for mTOR inhibition, sestrin2 may also carry the guanosine nucleotide dissociation inhibition (GDI) function. However, mutation studies of Arg419/Lys422/Lys426 in Sesn-C suggested that whether these amino acid residues are truly critical for GDI functions is still in question [35].

The availability of amino acids is critical for the regulation of protein synthesis in living organisms. Leucine, one of the essential amino acids, is indispensable for this process and, more importantly, leucine was found to be crucial for mTORC1 activation in cells [45]. Located in the Sesn-C of hSesn2 (Figure 2A), charged residues Glu451 and Arg390, from two sides of a single binding pocket, anchor leucine in place through salt bridges with the free amine and carboxyl groups, respectively, whereas the isopropyl side chain of the bound leucine forms extensive hydrophobic interactions with residues Leu389, Trp444, and Phe447 in the pocket. In addition to contacting the charged sides and hydrophobic base of the pocket, three threonine residues (Thr374, Thr377, and Thr386) are positioned directly above the leucine to form a “lid” that encloses the top of the leucine, thereby locking the ligand in place [36]. As a leucine sensor, sestrin2 inhibits mTORC1 activity through the Rag guanosine triphosphatases (GTPase) and its regulators-GATOR1 and GATOR2. Thus, the binding of leucine with sestrin2 disrupts the connection of sestrin2 with GATOR2, allowing GATOR2 to enhance mTORC1 activity [36]. It has previously been demonstrated that adult sestrin2 gene knockout mice subject to a fasting/refeeding regimen or maintained with a high-fat diet suffered from various metabolic derangements, such as hepatosteatosis, insulin resistance, and glucose intolerance, with increased ROS extent and mTORC1 activity [38,46].

Despite the availability of the crystal structure of hSesn2, the detailed molecular information for sestrin1 and sestrin3 remains to be fully elucidated. However, sequence alignment of the three human sestrins revealed an overall 44.8% amino acid sequence identity [47]. Furthermore, the amino acid residues critical for alkyl hydroperoxidase activity (Cys125, His132, and Tyr127), GATOR2-binding and AMPK activation for mTORC1 inhibition (Asp406 and Asp407), and leucine-binding (Glu451 and Arg390; Leu389, Trp444, and Phe447; Thr374, Thr377, and Thr386) are all evolutionarily conserved in the three human sestrins. It is therefore reasonable to speculate that hSesn1 and hSesn3 may share most, if not all, of the functional roles of hSesn2. However, as compared with sestrin2, the potential involvement of sestrin1 and sestrin3 in nervous systems has been studied much less well. Below, in Figure 2B, is the list of known biological functions of all three sestrins.

Expression of the sestrin2 genes is regulated by several critical transcription factors, enabling the cells to cope with various stressful insults. Initially the crucial role of the p53 tumor suppressor in regulating the expression of sestrin2 under hypoxic and genotoxic stress was revealed [33]. Later, additional studies revealed further transcription factors that are critical for the expression of sestrin2 under a variety of stressful conditions. Oxidative stress can activate the nuclear factor erythroid 2-related factor-2 (Nrf2) to regulate sestrin2 expression [48,49]. Hypoxia may induce sestrin2 expression where hypoxia-inducible factor-1 (HIF-1) may play a certain role [33,50,51,52], although the detailed mechanism is not well understood. In our earlier study [53], we found that brain-derived neurotrophic factor (BDNF) induced sestrin2 expression, which required dimerization of nuclear factor-κB (NF-κB) subunits p65 and p50. Further, BDNF also enhanced production of nitric oxide (NO), formation of 3′,5′-cyclic guanosine monophosphate (cGMP), and activation of cGMP-dependent protein kinase (PKG). Indeed, BDNF induced nuclear translocation of PKG-1 and its direct interaction with p65/p50 to form a ternary complex, thereby leading to heightened NF-κB binding to the sestrin2 gene promoter with resultant upregulation of its mRNA and proteins [53]. Apart from PKG/NF-κB, BDNF has also been shown to induce sestrin2 in neurons by activating transcription factor-4 (ATF4) [54]. In another recent study [40], we also found that NF-κB and p53 are involved in Aβ-induced sestrin2 expression in primary cortical neurons. Additional regulatory mechanisms responsible for sestrin2 induction under various stressful or physiological conditions may emerge in the near future.

**Figure 3:**
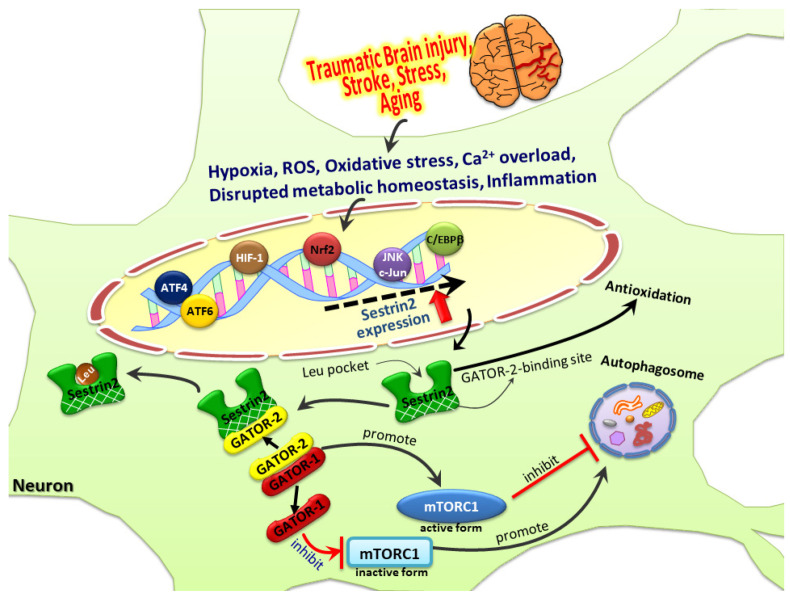
Brain trauma, stroke, neurological disorders, and aging induce hypoxia, the production of reactive oxygen species (ROS), Ca^2+^ overload, metabolic dyshomeostasis, and neuronal inflammation. Subsequently, the injury-induced signaling pathways promote sestrin2 expression via the activation of various transcription factors (which particular factors depending on which stressors), such as transcription factor-4 (ATF4), ATF6, hypoxia-inducible factor-1 (HIF-1), nuclear factor erythroid 2-related factor-2 (Nrf2), c-Jun N-terminal kinase (JNK)/c-Jun, and CCAAT-enhancer-binding protein-beta (C/EBPβ). Sestrin2, as a sensor for essential amino acids with a leucine-binding pocket, also has a binding site for the GTPase-activating protein activity toward Rags-2 (GATOR2). In the presence of sufficient amino acids available for protein synthesis, sestrin2 may bind to leucine and release the bound GATOR2. The freed GATOR2 can then physically associate with GATOR1, which can no longer bind to, and hence inhibit, mTORC1, thereby promoting protein synthesis while inhibiting autophagy. Under the stressful condition in which amino acids are insufficient, binding of GATOR2 to sestrin2 allows GATOR1 to inhibit mTORC1, thereby promoting autophagy while inhibiting protein synthesis. In addition to regulating autophagy and protein synthesis via binding with leucine or GATOR2, the endogenous alkyl hydroperoxidase activity of sestrin2 also exerts direct antioxidative actions.

Nutrients including amino acids, lipids, and glucose are crucial for the biosynthetic processes in the cell. An inadequate supply of nutrients can seriously modify cellular metabolism. Sestrin2 activation may serve as one of the metabolic accommodations to nutrient deficiency in cells [38]. Glucose starvation, inhibition of glycolysis, and impairment of mitochondrial respiration can disrupt energy production, leading to the activation of two transcription factors, ATF4 and Nrf2, that can bind directly to the consensus sequences within the promoter to induce sestrin2 gene transcription [49,55,56,57]. ATF4 is also involved in the induction of sestrin2 as a result of a deficiency in amino acid supply in mouse embryonic fibroblasts [58]. The inadequacy of growth factors may result in the expression of sestrin2. It has been demonstrated in cancer cells that serum deprivation can activate the c-Jun N-terminal kinase (JNK) pathway and upregulate sestrin2 expression, which could be abolished by specific siRNAs against JNK1/2 or c-Jun [59]. Various physiological and pathological conditions, such as excessive ROS generation, ischemia, Ca^2+^ dyshomeostasis, and inflammatory response can all cause an accumulation of misfolded proteins in the endoplasmic reticulum (ER), with resultant ER stress [60]. ER stress may lead to cellular dysfunction and/or cell death and contributes to the progression of many diseases. Modulation of ER stress pathways may represent a potential therapeutic strategy. It was reported that activating transcription factor-6 (ATF6)-dependent sestrin2 induction can lessen the severity of ER stress-mediated liver injury [61]. In another study, it was shown that the hepatoprotective role of sestrin2 against chronic ER stress depends on the regulation of CCAAT-enhancer-binding protein-beta (c/EBPβ) [62]. Together, these previous reports identify the crucial roles played by sestrin2 in dealing with various cellular stresses under diverse physiological and pathological conditions. A simplified diagram (Figure 3) demonstrates that distinct transcription factors are activated under a variety of stressful conditions, thereby leading to induction of sestrin2 expression, which can regulate autophagy and contribute to antioxidation.

## 3. Sestrin2 in Age-Related Clinical Conditions

Persuasive evidence supports the notion that aging is related to various harmful mechanisms, such as escalation of oxidative stress, instability of genetic materials, declined protein homeostasis, impaired mitochondrial function, increased cellular senescence, and stem cell exhaustion [63]. The accumulation of various cellular damages among tissues in aging organisms leads eventually to functional breakdown, causing disability or death. Therefore, aging is believed to be a risk factor for various disorders, such as cardiovascular diseases, stroke, type II diabetes, cancers, and neurodegenerative diseases [63,64,65]. Inhibition of either ROS production or mTORC1 activation may counteract aging [35], and as sestrin2 is characterized by both these functions, it may exert such beneficial effects [66,67]. In fact, enhancement of sestrin2 expression reduces aging markers. Conversely, lessening sestrin2 expression accelerates aging processes [68].

Aging is a predetermined time-related deterioration in various physiological conditions, and is a critical risk factor for cancer development. Cancer and aging involve similar processes of progressive time-dependent cellular damage. As sestrin2 is critically involved in aging [38,67], it may play a pivotal role in cancer progression, and is regarded as a potential tumor suppressor. In non-small cell lung cancer patients, higher sestrin2 expression was a favorable prognostic factor, while lower sestrin2 expression was accompanied by poor tumor cell differentiation, as well as more advanced staging in terms of tumor, node, and metastasis (TNM) [69]. It was shown that colorectal cancer patients with lower expression of sestrin2 showed poor prognostic outcomes [70]. Docosahexaenoic acid (DHA) can increase oxaliplatin-induced autophagic cell death through the ER stress/sestrin2 pathway in colorectal cancer [71], whereas downregulation of sestrin2 can accelerate colon carcinogenesis [72].

Hypernutrition, causing obesity, hepatosteatosis, and insulin resistance, is related to chronic activation of p70S6 kinase and mTORC1 [73]. Activation of sestrin2 can lower the extents of fatty liver and insulin resistance [73]. Sestrin2 can activate AMPK, inhibit mTORC1 activity, and maintain a high AKT level to suppress the extent of gluconeogenesis in the liver, thereby reducing the level of blood sugar. Sestrin2-deficient obese mice were found to present an evident decline of AKT activity, leading to insulin resistance and a higher level of glucose production [73]. In a recent study, serum levels of sestrins are significantly decreased in patients with diabetes and dyslipidemia. It appears that sestrin2 levels are robustly associated with diabetes, dyslipidemia, atherosclerosis, and the atherogenic index [74]. Declined serum sestrin2 levels were also observed in diabetic patients with nephropathy, particularly in those with macroalbuminuria [75].

It was demonstrated previously that loss of dSestrin (the only one sestrin homologue in Drosophila) results in age-associated pathologies, including cardiac dysfunction, muscle degeneration, and triglyceride accumulation. The cardiac dysfunction showed reduced heart rate and compromised heart function. The detrimental effects induced by dSestrin deficiency were generally inhibited by AICAR and rapamycin, the AMPK activator and the mTORC1 inhibitor, respectively [67]. These results indicate that the sestrin family may play crucial roles in the pathophysiology of cardiac regulation [76]. In a recent review article, sestrin2 is considered a rising star among antioxidants, with future therapeutic potential for reducing heart injury induced by oxidative stress, promoting cell survival through the activation of Nrf2/AMPK, and inhibiting mTORC1 to combat various cardiovascular diseases, such as cardiomyopathy, heart failure, and myocardial infarction [77]. Despite these promises, however, the occurrence of major adverse cardiac events is predicted in patients with chronic heart failure who have higher plasma sestrin2 concentrations [78]. The conflicting results as far as the beneficial or detrimental effects of sestrin2 in heart failure are concerned await further clarification.

Stroke is the most common age-related cerebral vascular disease and the chief cause of physical and intellectual disability in adults, as well as the leading cause of mortality in developed countries [79]. Several studies have investigated the roles of sestrin2 in cerebral ischemia [80,81,82,83]. It was demonstrated that sestrin2 can activate the Nrf2/heme oxygenase-1 (HO-1) pathway, leading to augmentation of angiogenesis following focal cerebral ischemia [82]. Another study also showed the critical role of sestrin2 in promoting angiogenesis in focal cerebral ischemia by activating the Nrf2/p62 pathway [81]. In contrast, silencing sestrin2 expression may reduce mitochondrial activity, suppress mitochondrial biogenesis, and ultimately exacerbate cerebral ischemia/reperfusion injury by preventing the AMPK/PGC-1α pathway [83]. Although sestrin2 seems to have pro-survival characteristics in the context of ischemic brain injury, the anti-inflammatory role of sestrin2 is unknown. In a recent study, it was demonstrated that sestrin2 exerts neuroprotective effects by changing microglial polarization and mitigating the extent of inflammation in the ischemic mouse brain, which may be due to the inhibition of the mTOR pathway and the restoration of autophagic flux [80]. It is to be expected that knowledge of the mechanisms underlying additional protective effects of sestrin2 may emerge in the not too distant future.

## 4. Potential Roles of Sestrin2 in Age-Related Neurodegenerative Diseases: Focusing on AD

As mentioned above, the sequences of the critical amino acid residues important for known biological activities of hSesn2, including alkyl hydroperoxide reductase, mTORC1 inhibition, and leucine binding, are also conserved in hSesn1 and hSesn3. However, the crystal structures of sestrin1 and sestrin3 are still not available. Nevertheless, there are a few studies implicating sestrin1 and sestrin3 in nervous system disorders. For example, sestrin1 may exert protective effects in oxygen-glucose deprivation/reoxygenation (OGD/R)-induced neuronal injury, a cellular model for mimicking cerebral ischemia/reperfusion injury in vitro [84]. Furthermore, sestrin3 has been identified as a pro-convulsant gene network in the human epileptic hippocampus [85]. Results derived from sestrin3 knockout rats also suggested that sestrin3 may increase the occurrence and/or severity of seizures [86]. Conversely, silencing rno-miR-155-5p in vivo mitigated the pathophysiological features associated with the status epilepticus, which was accompanied by attenuation of apoptosis in the hippocampus, by enhancing expression of sestrin3 in rats, implying that sestrin3 plays a beneficial role in offsetting temporal lobe epilepsy [87]. Further dissection of the pathophysiological roles of sestrin1 and sestrin3 will require a greater understanding of their molecular structures, as well as the upstream regulatory mechanisms involved in their expression in nervous systems.

Among age-related disorders, chronic neurodegenerative diseases are particularly concerning due to the lack of efficacious treatments, their irremediable clinical course, and their association with substantial social-economic burdens [1,2,3]. The potential roles of sestrin2 in combatting neurodegenerative diseases, including AD, Parkinson’s disease (PD), and Huntington’s disease (HD), while still awaiting further evidence, have gradually been recognized in recent years.

It is widely accepted that maintaining proper levels of reactive nitrogen species and ROS are crucial for ensuring regular neuronal function [88]. Yet, excessive ROS generation with heightened levels of oxidation in lipids, proteins, and DNA, or inherent lower antioxidant competence in the brain, may have detrimental effects on the organism and play a role in the pathophysiology of various chronic neurodegenerative diseases, including AD, PD, and HD [89,90]. Numerous mechanisms underlie oxidative stress-mediated neurodegeneration; these include calcium overload, glutamate excitotoxicity, inflammation, functional impairment of mitochondria, and apoptotic processes [88]. The ability to lessen these harmful effects may be the key to developing effective treatments for neurodegenerative diseases.

As mentioned above, sestrin2, with its dual functions, can directly reduce oxidative stress through restoring overoxidized peroxiredoxins, and indirectly lessen oxidative stress through regulating mTOR to augment the activity of autophagy, or specifically, mitophagy, to remove the worn-out or damaged mitochondria with higher levels of electron leakage and hence free radical production. The N-terminal domain of sestrin2 decreases oxidative stress by its helix-turn-helix motif, while the C-terminal domain of sestrin2 may physically associate with GATOR2, thereby causing the inhibition of mTORC1 [35]. Apart from the effect of oxidative stress, one more common pathogenic mechanism in chronic neurodegeneration is the deposition of aberrant and/or misfolded proteins, such as Aβ and tau protein in AD, Lewy body (LB) in PD, and mutant huntingtin in HD. Enhancing the activity of autophagy may help to eradicate neuronal dysfunction induced by misfolded proteins, thereby opening an opportunity towards developing a new therapeutic strategy for treating neurodegenerative diseases [91]. The dual biological functions of sestrin2, with increasing antioxidative ability and autophagy-promoting activity to eliminate aggregated proteins and damaged mitochondria, give this molecule a unique position in protecting neurons against degeneration.

PD is the second most common aging-related neurodegenerative disease that mainly presents syndromes with slow movements, tremors, and rigidity. The underlying cause of PD is not well understood but may involve various genetic and environmental factors [92]. The main pathological feature of PD is LB, which is composed of ubiquitin-bound, misfolded α-synuclein protein in the dopamine neurons in the substantia nigra of the midbrain [93,94]. In an in vitro PD model with 1-methyl-4-phenylpyridinium (MPP^+^), it was revealed that MPP^+^ neurotoxicity increases sestrin2 expression, whereas downregulation of sestrin2 with small interference RNA augments MPP^+^-related neurotoxicity in SH-SY5Y cells [95]. In another in vivo PD model induced by rotenone, sestrin2 exerts a protective effect over dopaminergic neurons against rotenone-induced neurotoxicity by activating an AMPK-dependent autophagy pathway [96]. In a clinical study, serum sestrin2 levels were found to be elevated in PD patients compared to controls [97]. In postmortem human samples, it was found that PD patients had higher expression levels of sestrin2 in the midbrain [95].

No report was available concerning HD and sestrin2 either in the clinical or pre-clinical studies. 3-Nitropropionic acid (3-NP) can inhibit the function of the mitochondrial respiratory complex II (also named succinate dehydrogenase), decrease ATP production, impair cellular energy metabolism, aggravate the extent of oxidative stress, cause mitochondrial DNA damage, and thus impair the function of mitochondria [98,99]. Although genetic models of HD are more popular due to their similarity to the phenotypes observed in HD, 3-NP is still a useful model to study neurotoxic phenomena, mitochondrial alterations, and neuroprotective effects for HD patients [100]. Therefore, 3-NP has been used as a pharmacological model to study neurodegeneration and neuronal death involving mitochondrial dysfunction in HD [101]. Despite the indirect relationship, we have shown that BDNF protects 3-NP-induced oxidative stress through augmenting sestrin2 expression. Furthermore, BDNF induction of sestrin2 implicates the NO/PKG/NF-κB pathway [53]. This study thus highlights the probable beneficial role of sestrin2 in this devastating hereditary neurodegenerative disease. Understanding the potential role of sestrin2 in impeding HD pathogenesis may require further investigation into the genetic models of HD, such as R6/2 or other knock-in mice.

AD is the most common age-related neurodegenerative disease involving various pathogenic mechanisms such as excitotoxicity, excessive generation of ROS, aberrant cell cycle reentry, impaired mitochondrial function, and DNA damage [15,16,17,18,19]. Although emerging roles of sestrin2 in various neurological diseases have been suggested before [102], limited studies concerning sestrin2 and AD have been reported [39,40,103,104,105,106,107]. In a 2003 study, in which human neuroblastoma CHP134 cells were analyzed with cDNA microarray technology with confirmation by semi-quantitative RT-PCR, it was revealed that sestrin2 is overexpressed under treatment of Aβ [107]. Furthermore, in human neuroblastoma SH-SY5Y cells, Aβ1-42 dose-dependently enhanced sestrin2 expression, whereas cotreatment with atorvastatin reversed sestrin2 back to the control level [103]. We have also demonstrated, in primary cortical neurons, that both Aβ25-35 and Aβ1-42 triggered the expression of sestrin2 [39,40], as is discussed in more detail below. In addition to these pre-clinical studies, the first human study reported in 2012 using postmortem brain tissues from advanced AD patients with immunohistochemistry findings showed intense sestrin2 expression in the neuropil, which may suggest a diffuse expression in various components among neurons, glia, and vascular cells. Using double-labeling immunofluorescence microscopy, co-localization between phosphorylated tau and sestrin2 is observed in the neurons and the neurites in neurofibrillary lesions [106]. These findings together implied that sestrin2 is expressed at least in the neurons of AD patients. Another clinical study demonstrated significant overexpression of sestrin2 protein and mRNA in the serum of AD patients as compared to the mild cognitive impairment (MCI) and the age-matched control groups. A difference in serum sestrin2 concentration between MCI and the control groups was also evident. However, no significant difference in sestrin1 levels was observed among the study groups. These results therefore suggested the potential role of sestrin2 as a biomarker in the analysis of peripheral blood in AD patients, and highlighted the importance of sestrin2, as opposed to sestrin1, in the progression of AD [104]. Despite these arguments supporting the important roles of sestrin2 in AD, it should be noted that, with similar biological functions and significantly conserved amino acid sequences identified across the different members of the sestrin family, although potential involvements of sestrin1 and sestrin3 in AD have not been reported, they certainly cannot be overlooked. Overall, this review has only focused on discussing the potential roles of sestrin2 in neurodegenerative disorders, AD in particular.

**Figure 4:**
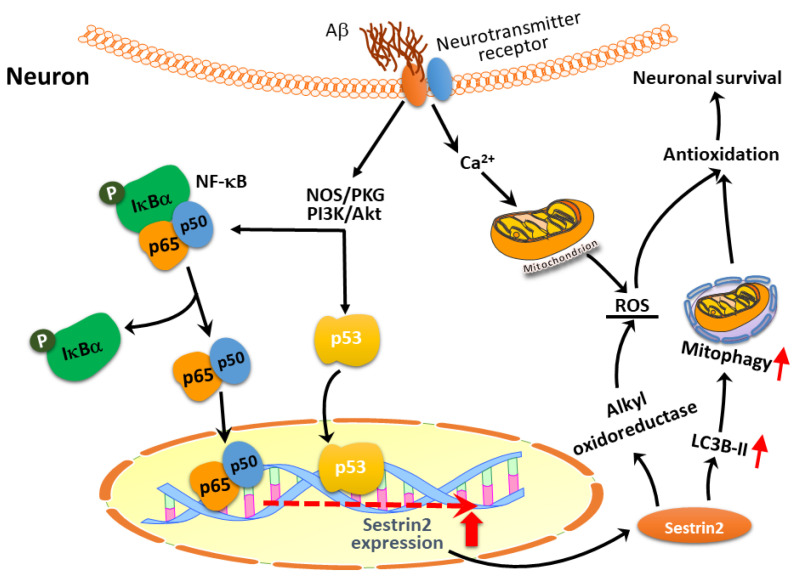
Amyloid-beta peptide (Aβ) enhances calcium dyshomeostasis and the generation of reactive oxygen species (ROS), thereby leading to oxidative stress with damaged mitochondria. Meanwhile, Aβ also induces p53, as well as nuclear factor-kappaB (NF-κB) subunits p65 and p50 via activation of nitric oxide synthase (NOS)/cGMP-dependent protein kinase (PKG) and phosphatidylinositol 3-kinase (PI3K)/Akt. The transcription factors, p50, p65, and p53 translocate into the nucleus of the neuron to promote expression of sestrin2 mRNA, as indicated by the red dashed arrow. The alkyl hydroperoxidase activity of sestrin2 may neutralize excessive ROS generated by Aβ with antioxidative functions. In addition, sestrin2 may trigger autophagy, as is indicated by the conversion of the microtubule-associated protein-1 light-chain 3B-I (LC3B-I) into LC3B-II, and possibly also mitophagy, in order to remove Aβ-damaged mitochondria known to produce more ROS. Sestrin2 thus may function as an endogenous protective mediator inducible by Aβ that contributes to neuronal survival against Aβ neurotoxicity.

We have explored the potential link between sestrin2 and Aβ-induced neurotoxicity [39,40]. In an in vitro study, we demonstrated that sestrin2 was induced by Aβs, including both Aβ25-35 and Aβ1-42, in primary culture of fetal rat cortical neurons. We further showed an in vivo result of increased sestrin2 expression in the aged APPswe/PSEN1dE9 transgenic mice. More importantly, sestrin2 functions as an endogenous protective moderator, through the adjustment of autophagy, against Aβ-induced neurotoxicity [39]. It is well known that sestrin2 has an antioxidant character and plays a critical role in age-related diseases [66]. In our recent report [40], Aβ-induced sestrin2 expression in primary cortical neurons was found to have an antioxidant effect, resulting in the suppression of Aβ-mediated ROS production, enhancement of lipid peroxidation, and formation of 8-hydroxy-2-deoxyguanosine (8-OH-dG) as an index of oxidative DNA damage. Interestingly, we found that lentivirus-mediated overexpression of the N-terminal domain of sestrin2 in primary cortical neurons completely blocked Aβ25-35-induced ROS production, whereas overexpression of the C-terminal domain partially, but statistically significantly, suppressed ROS formation. Although the sestrin2 C-terminal domain is known to have the capability of inhibiting mTORC1 to promote autophagy [35], we speculated that augmentation of autophagy with enhanced removal of damaged mitochondria, or mitophagy, may also contribute to the antioxidant function of sestrin2. Upstream of sestrin2, we found that the observed Aβ effect on sestrin2 expression is at least partially mediated by p53 and NF-κB. Indeed, apart from regulating sestrin2 induction, p53 and NF-κB subunits p65/p50 also affect the expression of each other [40]. Furthermore, upstream of p53 and NF-κB, we identified at least two signaling pathways, namely nitric oxide synthase/cGMP-dependent protein kinase (NOS/PKG) and phosphatidylinositol 3-kinase (PI3K)/Akt, that may have contributed to the observed Aβ induction of sestrin2 in cortical neurons [40]. A diagram summarizing our findings is shown in Figure 4, below.

The synaptic activity of neurons can affect the homeostasis of Aβ and tau. Both are aggregated and accumulated during the progression of AD and are critical for neuronal function. Furthermore, impairment of synaptic activity is linked with AD [108]. Physiologic synaptic activity, through NMDA receptor signaling, can enhance antioxidant activity and increase sestrin2 expression to exert a protective effect through transcription factor C/EBPbeta [109]. Presenilin proteins are catalytic components of γ-secretase involved in various functions such as proteolytic cleavage of the Notch and APP, adjustment of neurotransmitter release, and are vital for the survival of neurons in aging [110]. Mutations of the presenilin genes are one of the main causes of familial AD [111]. Impairment of presenilin activity may compromise synaptic functions, resulting in neurodegeneration and ultimately dementia [112]. It was demonstrated that cells deficient in presenilin have lower levels of sestrin2 and are accompanied with mTORC1 dysregulation. These findings show that sestrin2, through attenuation of oxidative stress and its nutrient-sensing ability via mTOR, plays a critical role in AD-related conditions [105].

Emerging evidence suggested the potential benefit of sestrin2 in AD. Medications with the capability to alter sestrin2 expression may therefore have the potential to prevent or delay the clinical deterioration of this neurodegenerative disease. It was previously shown that atorvastatin reduces Aβ-induced synaptotoxicity and memory impairment through a p38MAP kinase pathway [113]. Atorvastatin could also activate autophagy through AMPK/mTOR signaling [113,114]. In a recent study, it was demonstrated that sestrin2 and the autophagy marker LC3II were increased with Aβ treatment in human neuroblastoma cells; co-treatment of atorvastatin and Aβ reduced oxidative stress and decreased sestrin2 expression [103]. We have shown before that BDNF can induce sestrin2 expression in rat primary cortical neurons and exert a protective effect against 3-NP neurotoxicity by reducing the production of free radicals [53]. BDNF is known to protect against Aβ-induced neurotoxicity in vitro as well as in rodent and primate models [115,116]. However, whether sestrin2 induction by BDNF contributes to this neuroprotective effect has not been tested. The possibility certainly cannot, however, be excluded.

**Figure 5:**
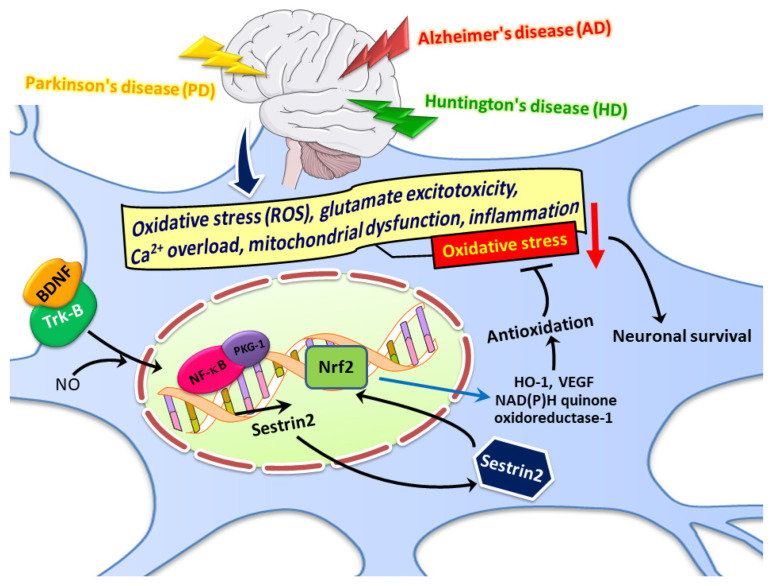
Multiple pathogenic mechanisms including oxidative stress, with excessive production of reactive oxygen species (ROS), glutamate-induced excitotoxicity, calcium overload, mitochondrial dysfunction, and inflammation contribute to neuronal death in various neurodegenerative disorders like Alzheimer’s disease (AD), Parkinson’s disease (PD), and Huntington’s disease (HD). Brain-derived neurotrophic factor (BDNF) enhances sestrin2 expression via signaling pathways involving nitric oxide (NO)/3′,5′-cyclic guanosine monophosphate (cGMP)-dependent protein kinase-1 (PKG-1)/nuclear factor-kappaB (NF-κB). In addition to the alkyl hydroperoxidase activity and autophagy promotion, sestrin2 may also have antioxidant properties by activating nuclear factor erythroid 2-related factor-2 (Nrf2) with enhanced expression of antioxidant proteins like heme oxygenase-1 (HO-1), vascular endothelial growth factor (VEGF), and NAD(P)H quinone oxidoreductase-1. These antioxidant proteins then mitigate oxidative stress, as indicated by the red arrow, that is commonly observed in various neurodegenerative diseases. The possibility that BDNF may exert its neuroprotective effects, in addition to its well-known neurotrophic actions, via induction of sestrin2 in various neurodegenerative disorders, requires further investigation.

In addition to alkyl hydroperoxidase activity and enhanced autophagy to alleviate oxidative stress, sestrin2 may also trigger the Nrf2/ARE pathway to augment antioxidant responses. For example, following photochemical cerebral ischemia in rats, expression of sestrin2, Nrf2, HO-1, and VEGF were significantly increased. Overexpression of sestrin2 by AAV injection further enhanced their expression [82]. In another study of photothrombotic ischemia in rats, sestrin2 may promote angiogenesis by activating Nrf2 via upregulation of p62 with enhanced interaction between p62 and Keap1, thereby improving the neurological function, reducing brain infarction, and alleviating brain edema [81]. Sestrin2 was also a direct target of microRNA miR-148b-3p in the HT22 hippocampal neurons challenged with OGD/R. Furthermore, Nrf2/ARE was a downstream antioxidant signal contributing to the observed protective effects through miR-148b-3p inhibition, and hence sestrin2 induction, in response to OGD/R injury [117]. In the H_2_O_2_-stimulated retinal ganglion cells (RGCs), sestrin2 overexpression increased the nuclear translocation of Nrf2, thereby upregulating the Nrf2/ARE target genes, including HO-1 and NAD(P)H quinone oxidoreductase-1 [118]. As mentioned above, sestrin2 itself may be a downstream target of Nrf2 [48,49]. Although these studies were conducted in non-neuronal cells like mammary epithelial cells and hepatocytes, the possibility that Nrf2 activation may induce sestrin2 expression in the nervous system cannot be excluded. Whether sestrin2 may trigger its own expression, thereby forming a positive feedforward loop, via Nrf2/ARE in neurons, also requires further investigation. The potential role of sestrin2 in age-related neurodegenerative diseases is demonstrated in Figure 5.

## 5. Medications or Chemical Compounds Capable of Altering Sestrin2 Expression

The outcomes of clinical trials using drugs to target amyloid and tau have been unsatisfactory up to now, thereby leading to enthusiasm in targeting alternative mechanisms in AD studies [119,120]. Drug repurposing involves taking the research into an existing, ready-to-use drug and assessing its therapeutic potential with respect to another disease [121,122]. Several well-known success stories include aspirin, sildenafil, and thalidomide [123]. This approach may provide a less expensive and quicker method of drug discovery. Several recent review articles emphasize the clinical potential of drug repurposing in the context of AD [120,124,125,126]. It would be worthwhile to search among medications with neuroprotective effects, as these are likely to have a better chance of achieving clinically meaningful results with neurodegenerative diseases [127]. The potential of certain medications to activate sestrin2 expression requires further investigation.

Several studies revealed that certain drugs capable of activating sestrin2 expression in various disease models may be worth testing in AD as well. It was shown that empagliflozin, which is a sodium-glucose cotransporter 2 (SGLT2) inhibitor useful for treating diabetes mellitus (DM) patients, can regulate sestrin2, the AMPK-mTOR pathway, and ROS homeostasis to improve obesity-related cardiac dysfunction in mice [128]. Another study demonstrated that liraglutide, a glucagon-like peptide 1 (GLP-1) agonist for DM patients, may lessen obesity-related fatty liver disease through regulating the sestrin2-mediated Nrf2/HO-1 pathway [129].

5-Fluorouracil is an antimetabolite widely used for chemotherapeutic treatment of cancers [130,131]. It was shown that 5-fluorouracil increases sestrin2 levels in a p53-dependent pathway and inhibits cancer cell migration in an in vitro colon cancer study [132]. Nelfinavir, an ER stress-inducing agent, and bortezomib, a proteasome inhibitor, can both enhance sestrin2 expression, which may be useful to treat cancers [133]. Interestingly, nelfinavir inhibited endogenous Aβ1-40 production from primary cultured human cortical neurons [134]. Whether these reagents may also carry therapeutic potential for AD requires further investigation.

Other chemical compounds such as resveratrol and melatonin possessing pleiotropic effects like antioxidancy or anti-inflammation were studied based on their capability of upregulating sestrin2 in various disease models [135,136,137]. Resveratrol is a naturally occurring polyphenol that is abundant in grape seeds and skin [138,139]. It can offer protective effects against various age-related diseases like AD through diverse mechanisms [138,140]. These molecular mechanisms include modulation of NF-κB, regulation of inflammatory cytokines, production of antioxidant enzymes, angiogenesis, apoptosis, lipid metabolism, and mitochondrial biogenesis-all critical for its potential clinical application [141,142]. It was demonstrated before that resveratrol affects sestrin2 gene induction and inhibits liver X receptor-alpha (LXRα)-mediated hepatic lipogenesis [137]. Methylglyoxal is implicated in the formation of advanced glycation end-products associated with diabetes and age-related neurodegenerative diseases [143]. In a previous study using methylglyoxal to induce cell death in HepG2, a human liver cancer cell line, it was found that resveratrol reduces methylglyoxal-induced mitochondrial impairment and apoptosis through sestrin2 induction [136]. Other flavonoid polyphenols or flavone derivatives, such as eupatilin [144,145], pentamethylquercetin [146], and isorhamentin [147], also possess the capability to alter sestrin2 expression and are worth studying further in AD models.

Melatonin, a molecule widely distributed in living organisms, is involved in various physiological and biological functions among diverse tissues and organs. It possesses prominent antioxidant effects, functions as a free radical scavenger, augments antioxidant enzymes, lessens mitochondrial electron leakage, and reduces pro-inflammatory signaling pathways [148]. These properties of melatonin underline the possibility for future clinical use in numerous disorders, including neurodegeneration [149]. It was shown that melatonin can inhibit proliferation and apoptosis in the vascular smooth muscle through upregulation of sestrin2, which may be important in preventing atherosclerosis and restenosis of vessel lumen [135]. It would be interesting to know the effect of sestrin2 expression under melatonin treatment in a stressful condition, such as in Aβ-induced neurotoxicity.

It is believed that a long list of medications, natural products, chemical compounds, or small molecules capable of altering sestrin2 expression may exert beneficial effects over AD-related mechanisms. This awaits further investigation and may lead to more opportunities for treating such devastating neurodegenerative diseases as AD.

## 6. Conclusions and Future Perspectives

Being a member of the sestrin family, sestrin2 acts as a crucial intracellular detector capable of regulating various biological processes to maintain the homeostasis of living organisms. Emerging evidence reveals that sestrin2 may have beneficial effects for vulnerable cells, such that they may adapt to numerous pathological situations under diverse stressful conditions, including DNA injury, hypoxic state, metabolic dyshomeostasis, and oxidative stress. In age-related neurodegenerative disorders, excessive generation of ROS and dysfunction of autophagy may play pivotal roles in the pathogenesis among these diseases. Sestrin2, with distinctive dual-functional sites to counteract excessive ROS generation and inhibit mTOR activity for autophagy promotion, is presumed to play a crucial role in AD, although at present only limited information is available to firmly establish this notion. Certain medicinal compounds or natural products, such as flavonoid-related products, can alter the expression levels of sestrin2. It is believed that any means of increasing sestrin2 expression may possess significant clinical implications for the abatement of AD-related neurodegeneration. The possibility awaits further investigation. It is uncertain, however, whether the overactivation of sestrin2 may result in detrimental effects due to autophagic dysfunction. It may be difficult to determine the pros and cons of excessive activation or inhibition of autophagy in terms of neurodegenerative diseases, including AD. This concern further reveals the crucial need for a thorough understanding of both the downstream targets, as well as the upstream regulators, of sestrin2. Fuller elucidation of the signaling pathways of sestrin2 would accelerate the discovery of novel therapies for disease treatment, especially for those diseases with a devastating clinical course, such as AD.
